# The Food of a Human Being

**Published:** 1869-11

**Authors:** 


THE FOOD OF A HUMAN BEING.—The following estimate of "what goes in at the mouth" of an epicure, is startling, if true: From an article recently published we extract the following estimate of what a noted English epicure ate during the 70 years that he lived: 10 oxen, 200 sheep, 100 calves, 200 lambs, 50 pigs, 1,200 fowls, 300 turkeys, 150 geese, 400 ducks, 260 pigeons, 1,400 partridges and quail, 600 woodcock, 1,400 snipe and other small game, besides 500 hares and rabbits, 40 deer, 120 guinea fowls, 10 peacocks, and 260 wild fowls. In fish, 100 turbots, 140 salmon, 220 cod, 260 trout, 400 mackerel, 400 flounders, 200 eels, 150 haddock, 400 herrings, and 10,000 smelts; also 20 turtles, 30,000 oysters, 3,500 lobsters and crabs, 300,000 prawns, shrimps, sardines and anchovies. In fruits, 150 pounds grapes, 50 pineapples, 2,000 peaches, 1,400 apricots, 240 melons, and some hundred thousand plums, green-gages, apples, and pears, and millions of cherries, strawberries, currants, walnuts, chestnuts, figs, almonds, etc. In vegetation of other kinds, 25,575 pounds weight; about 2,334 pounds of butter; 684 pounds of cheese; 21,000 eggs; bread, 14,000 pounds; of salt and pepper, 1,000 pounds; of sugar, 4,500 pounds. In liquids, 49 hogsheads of wine, 1,394 gallons of beer; 584 gallons of spirits, 5,394 gallons of coffee, cocoa and tea, 1,364 gallons of milk, and 2,736 gallons of water.—*Phil. Med. and Surg. Reporter.*

How did the teeth stand it *Mr. Reporter?*—ED.

